# Identification and genetic counseling for a novel variant of *MLH1* associated with lynch syndrome in colorectal cancer: a case report

**DOI:** 10.1093/gastro/goad049

**Published:** 2023-08-23

**Authors:** Xiaohuan Lu, Hongyan Zhang, Luming Xu, Yang Cao, Yuan Li, Wei Li, Gang Li, Feng Xue, Zheng Wang

**Affiliations:** Department of Gastrointestinal Surgery, Union Hospital, Tongji Medical College, Huazhong University of Science and Technology, Wuhan, Hubei, P. R. China; Department of Clinical Laboratory, Union Hospital, Tongji Medical College, Huazhong University of Science and Technology, Wuhan, Hubei, P. R. China; Department of Clinical Laboratory, Union Hospital, Tongji Medical College, Huazhong University of Science and Technology, Wuhan, Hubei, P. R. China; Department of Gastrointestinal Surgery, Union Hospital, Tongji Medical College, Huazhong University of Science and Technology, Wuhan, Hubei, P. R. China; Department of Gastrointestinal Surgery, Union Hospital, Tongji Medical College, Huazhong University of Science and Technology, Wuhan, Hubei, P. R. China; Department of Gastrointestinal Surgery, Union Hospital, Tongji Medical College, Huazhong University of Science and Technology, Wuhan, Hubei, P. R. China; Department of Gastrointestinal Surgery, Union Hospital, Tongji Medical College, Huazhong University of Science and Technology, Wuhan, Hubei, P. R. China; Department of General Surgery, Affiliated Hospital of Yangzhou University, Yangzhou, Jiangsu, P. R. China; Department of Gastrointestinal Surgery, Union Hospital, Tongji Medical College, Huazhong University of Science and Technology, Wuhan, Hubei, P. R. China

## Introduction

Lynch syndrome (LS) is an autosomal dominant condition caused by pathogenic variants in mismatch repair (MMR) genes. LS is associated with an 80% lifetime risk for colorectal cancer (CRC); it is also characterized by extracolonic tumors, including endometrial, stomach, or ovarian cancer [1, 2].

The clinical diagnosis of LS generally relies on the Amsterdam or Bethesda criteria. However, the sensitivity or specificity of these two criteria is limited mainly due to the lack of accurate family history information [3]. With advances in technology, conditions for diagnosis and treatment of LS have greatly improved. Germline genetic testing of pathogenic variations of the MMR genes has been applied to diagnose LS in recent years [4]. Meanwhile, with growing knowledge of LS, more germline variants have been found for molecular screening of LS.

Here, we report a novel heterozygous pathogenic germline variant of *MLH1*, c.482delC (p.Thr161ArgfsTer6) that was identified in a Chinese LS family. This work provides guidance for LS diagnosis and genetic counseling.

## Case report

**Figure 1.**
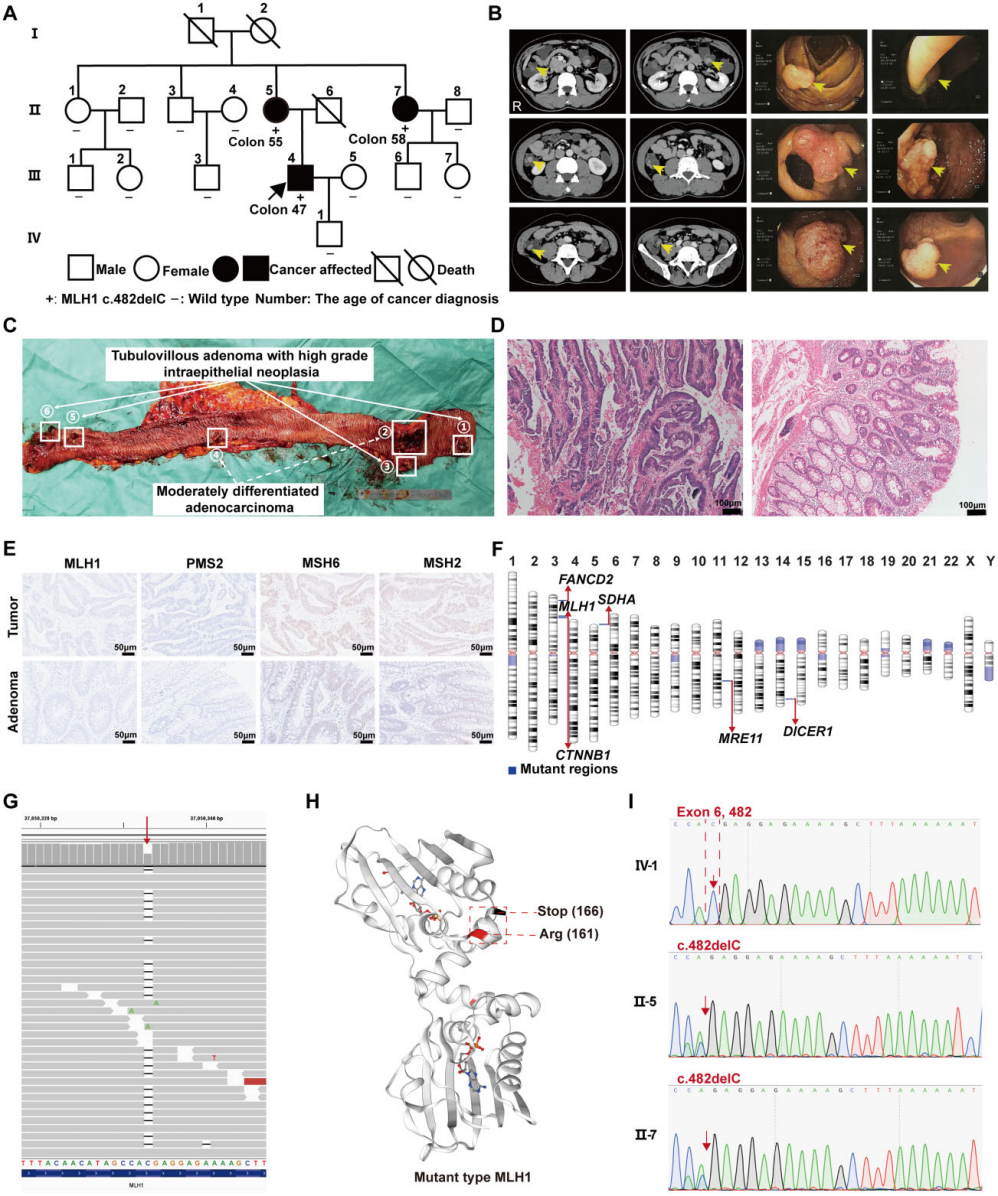
Clinical and molecular analysis of the LS family. (A) Pedigree structure of the LS family. The arrow indicates the proband (III-4). (B) Enhanced CT and electronic colonoscopy images of the proband (III-4). (C) Image of tumor specimen after subtotal colectomy of the proband (III-4). Arrows indicate adenocarcinoma and adenoma. (D) H&E staining of tumor and polyp tissues. (E) IHC staining for MMR proteins in the tumor and polyp tissues. (F) Landscape of gene mutations. (G) *MLH1* c.482delC was shown by using the Integrative Genomics Viewer (IGV). (H) 3D structure modeling of mutant MLH1 protein. (I) Sequencing chromatograms of *MLH1* exon 6 regions in affected members. Scale bars: 100 and 50 μm.

In this LS family, three members (II-5, II-7, and III-4) had colon cancer history (Figure 1A). The proband (III-4), a 47-year-old man, was admitted to hospital and treated for abdominal distension discomfort and hematochezia. Laboratory examinations showed a significantly decreased hemoglobin level of 108 g/L (internal reference, 130–175 g/L). Abdominal enhanced computed tomography (CT) scans indicated segmental proximal intestinal wall thickening in the sigmoid colon and showed scattered soft tissue dense nodules (polyps likely) in both the colon and the rectum (Figure 1B). Then, six polyps in the colon were identified by using colonoscopy (Figure 1B) and pathological biopsy revealed adenoma with low-grade and partial high-grade intraepithelial neoplasm. Another two colon cancer members (II-5 and II-7) were both treated with laparoscopic colorectal resection. The proband’s mother (II-5) was diagnosed with sigmoid colon cancer (pT3N1M0) at 55 years old with no multiple colorectal polyps and his aunt (II-7) was diagnosed with ascending colon cancer (pT2N0M0) at 58 years old. The other family members had no history of cancer or gastrointestinal polyposis.

The proband was treated with laparoscopic subtotal colectomy. Gross examination showed that a giant ulcerated tumor (7 cm × 3.5 cm) and a bulging tumor (1.6 cm × 1.1 cm) in the resected colon (Figure 1C); histopathological analysis revealed moderately differentiated adenocarcinoma with partial mucinous adenocarcinoma invading the subserosa (Figure 1D). The other four polyps were tubulovillous adenomas with high-grade intraepithelial neoplasia (Figure 1D). No lymphovascular invasion (LVI), perineural invasion (PNI), or lymph node metastasis was discovered, and there was no cancer invasion in the resection margins. The proband was initially diagnosed as having T3N0M0 colon cancer and multiple adenomas in the colorectum.

Based on the family history, the overall predicted probability of *MLH1*, *MSH2*, *MSH6*, *PMS*2, or *EPCAM* mutation was ≥50% for the proband in the PREMM5 model (https://premm.dfci.harvard.edu/). Considering the overall predicted probability is ≥2.5%, referral for genetic evaluation, including microsatellite instability (MSI) or immunohistochemistry (IHC) testing, genetic counseling, and/or germline genetic testing, was recommended. The IHC staining of the colon tumor and adenoma polyps showed the loss of MLH1 and PMS2 expression (Figure 1E); the MSI testing report (2B3D NCI Panel) also demonstrated MSI-high in tumor tissues. Therefore, to further confirm this case as LS, we recommended germline genetic testing to the proband. A germline multigene panel test (MGPT) was performed for molecular analysis (Supplementary Tables 1 and 2). According to Human Genome Variation Society nomenclature, next-generation sequencing analysis identified a novel variant, NM_000249.4(MLH1):c.482delC (p.Thr161ArgfsTer6) heterozygous (Figure 1F and G), and the variant was pathogenic when using *in silico* analysis. Swiss-Model revealed that the mutant amino acid changed the protein transcription and translation, resulting in the loss of the *MLH1* mismatch repair function (Figure 1H). As a result, threonine was changed to arginine at position 161 and the downstream translation was terminated because of the frameshift-caused stop codon from position 161 to 166 (p.Thr161Argfs*6). We next performed Sanger sequencing for subsequent family cascade testing. Two family members (II-5 and II-7) were also identified to carry this pathogenic variant (Figure 1I). The other 11 family members, including the proband’s son (IV-1, 13 years old), did not harbor the variant or have LS phenotype.

The proband was finally diagnosed with LS with multiple colonic polyps. During the follow-up visit, he and the other two affected family members (II-5 and II-7) underwent endoscopic surveillance every 1–2 years and no polyp or tumor recurrence was found.

## Discussion

LS is the most common inherited cancer syndrome and accounts for 2%–3% of all CRC cases [5]. In the era of precision medicine, germline MGPT plays an important role in LS diagnosis. MGPT has a higher yield than single-gene testing or sequential single-syndrome testing for identifying individuals with a pathogenic variant in a cancer risk gene. It may allow additional opportunities for the early detection and prevention of cancer [6]. Here, we reported a CRC patient harboring a novel germline variant c.482delC (p.Thr161ArgfsTer6) of the *MLH1*; two members of the proband’s family (II-5 and II-7) also carried the *MLH1* pathogenic variant. This mutation is a novel pathogenic variant; it has not been previously documented in the International Society for Gastrointestinal Hereditary Tumors (InSiGHT) database and the ClinVar database.

During the genetic counseling period, the possible inherited cancer risk and surveillance recommendations were advised to the proband and his relatives. Even though the proband received a laparoscopic subtotal colectomy, he still had a 7%–44% cumulative risk of developing cancer in the future [7]. Therefore, a colonoscopy every 1–2 years was recommended for the proband as well as his mother and aunt with LS. Male CRC patients (age >40 years, having *MLH* pathogenic variants) may benefit from a shorter 1-year interval screening [8]. Additionally, his mother and aunt have a lifetime risk of ≤60% of suffering from endometrial cancer, the second most common cancer in women with LS [2]. Therefore, screening via endometrial biopsy every 1–2 years can be considered. In China, very few LS patients are accurately and molecularly diagnosed and optimally followed up as having a high risk of developing cancer. Therefore, more pathogenic variant carriers are expected to be detected early through a comprehensive screening strategy. As more studies have reported [9, 10], a better understanding of LS will be helpful to reduce mortality and strengthen the management of LS.

In summary, this report enriches the LS mutation spectrum and provides useful information for diagnosing and clinically managing LS patients.

## Supplementary Material

goad049_Supplementary_Data

## Data Availability

The raw sequence data reported in this paper have been deposited in the Genome Sequence Archive in National Genomics Data Center, China National Center for Bioinformation/Beijing Institute of Genomics, Chinese Academy of Sciences (GSA for Human: HRA002717) that are publicly accessible at https://ngdc.cncb.ac.cn/gsa.
