# Supplementary material for: Identification and genetic counseling for a novel variant of MLH1 associated with lynch syndrome in colorectal cancer: a case report
Source: Gastroenterol Rep (Oxf). 2023 Aug 23;11:goad049. doi: 10.1093/gastro/goad049 (PMC10448983; doi:10.1093/gastro/goad049)
Supplement: goad049_Supplementary_Data [file goad049_supplementary_data.docx]

**Supplementary Material**

**Supplementary Table 1.** **102 genes used in the** **multigene panel test.**

| *AKT1* | *CDK12* | *ERCC1* | *GALNT12* | *MSH3* | *POLE* | *RAF1* | *SUFU* |
| --- | --- | --- | --- | --- | --- | --- | --- |
| *APC* | *CDK4* | *ERCC2* | *GREM1* | *MSH6* | *POT1* | *RB1* | *TGFBR2* |
| *ATM* | *CDKN1B* | *ESR1* | *GSTP1* | *MUTYH* | *PPM1D* | *RECQL* | *TP53* |
| *ATR* | *CDKN2A* | *EZH2* | *HOXB13* | *NBN* | *PRF1* | *RECQL4* | *TSC1* |
| *AXIN2* | *CHEK1* | *FAM175A* | *KIT* | *NF1* | *PTCH1* | *RET* | *TSC2* |
| *BAP1* | *CHEK2* | *FANCA* | *KRAS* | *NF2* | *PTEN* | *SDHA* | *VHL* |
| *BARD1* | *CTNNA1* | *FANCC* | *MEN1* | *NTHL1* | *PTPN11* | *SDHAF2* | *WT1* |
| *BLM* | *CTNNB1* | *FANCD2* | *MET* | *NTRK1* | *RAD50* | *SDHB* | *XRCC1* |
| *BMPR1A* | *DICER1* | *FANCI* | *MITF* | *PALB2* | *RAD51B* | *SDHC* | *XRCC2* |
| *BRCA1* | *EGFR* | *FANCL* | *MLH1* | *PIK3CA* | *RAD51C* | *SDHD* | *YAP1* |
| *BRCA2* | *EMSY* | *FANCM* | *MLH3* | *PMS1* | *RAD51D* | *SMAD4* | *PPP2R2A* |
| *BRIP1* | *EPCAM* | *FH* | *MRE11* | *PMS2* | *RAD54B* | *SMARCA4* |  |
| *CDH1* | *ERBB2* | *FLCN* | *MSH2* | *POLD1* | *RAD54 L* | *STK11* |  |

**Supplementary Table 2.** **Germline gene variants of the family members.**

| Individual ID | Chr | Gene | Func.refgene | Nucleotide change | Amino acid change | Het/Hom | Prediction |
| --- | --- | --- | --- | --- | --- | --- | --- |
| Ⅲ-4 | Chr 3 | *FANCD2* | exon16 | c.1306C>A | p.Leu436Met | Het | VUS |
| Ⅲ-4 | Chr 3 | *CTNNB1* | exon4 | c.486C>T | p.Asp162Asp | Het | VUS |
| Ⅲ-4 | Chr 5 | *SDHA* | exon11 | c.1516A>G | p.Ile506Val | Het | VUS |
| Ⅲ-4 | Chr 11 | *MRE11* | exon13 | c.1418T>C | p.Leu473Ser | Het | VUS |
| Ⅲ-4 | Chr 14 | *DICER1* | exon21 | c.3518C>G | p.Thr1173Arg | Het | VUS |
| Ⅲ-4/Ⅱ-5/Ⅱ-7 | Chr 3 | *MLH1* | exon6 | c.482delC | p.Thr161Argfs*6 | Het | P |

Chr: chromosome; Het: homozygous; Hom: heterozygous; VUS: uncertain significance; P: pathogenic
